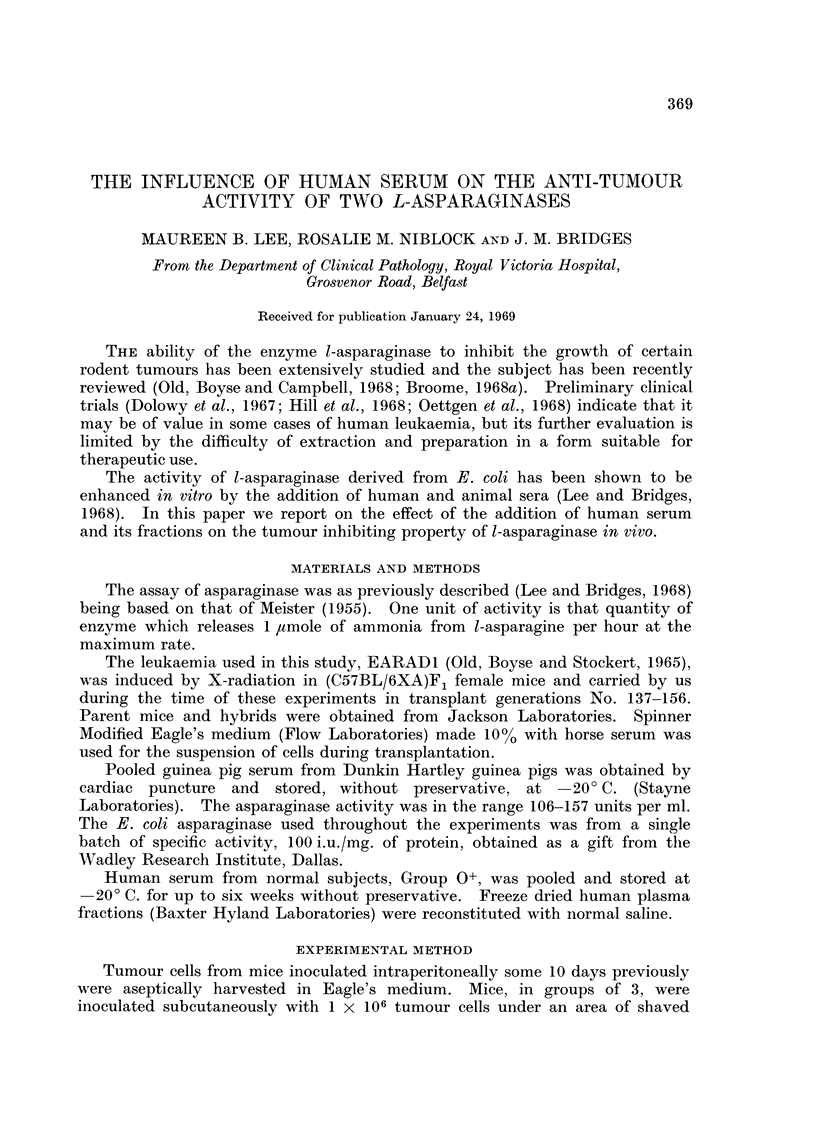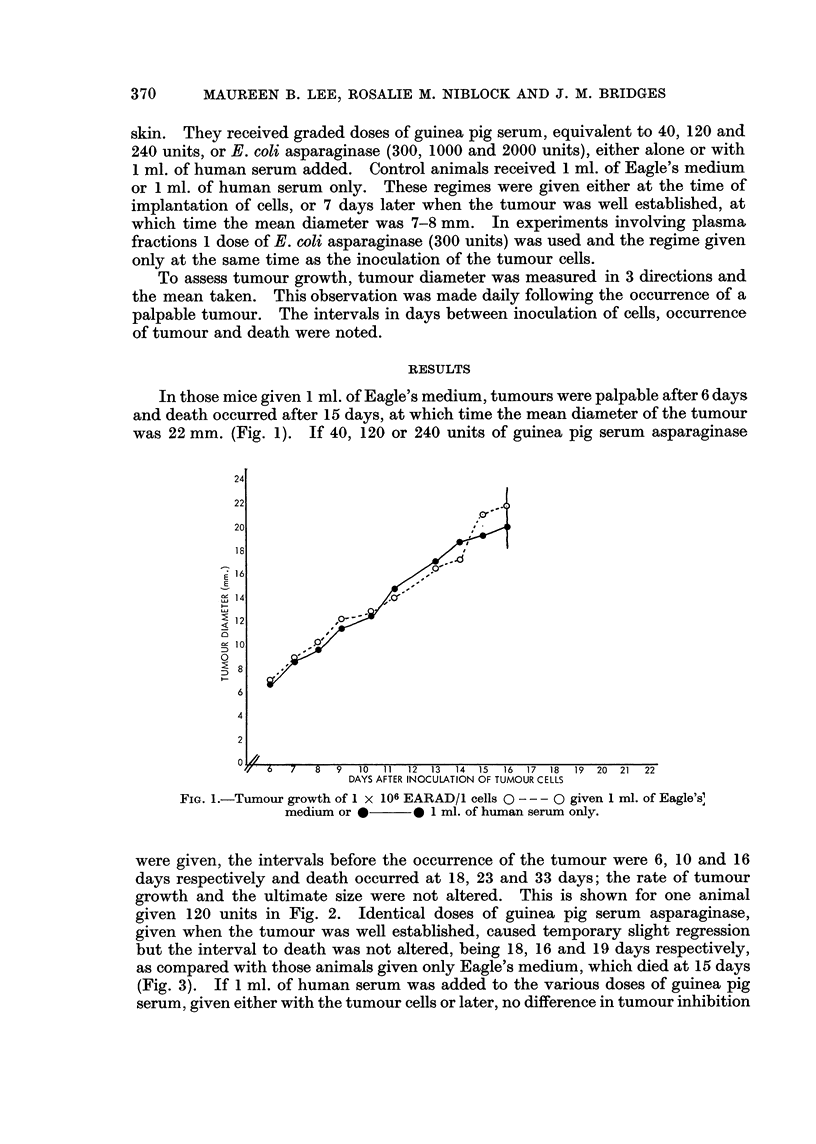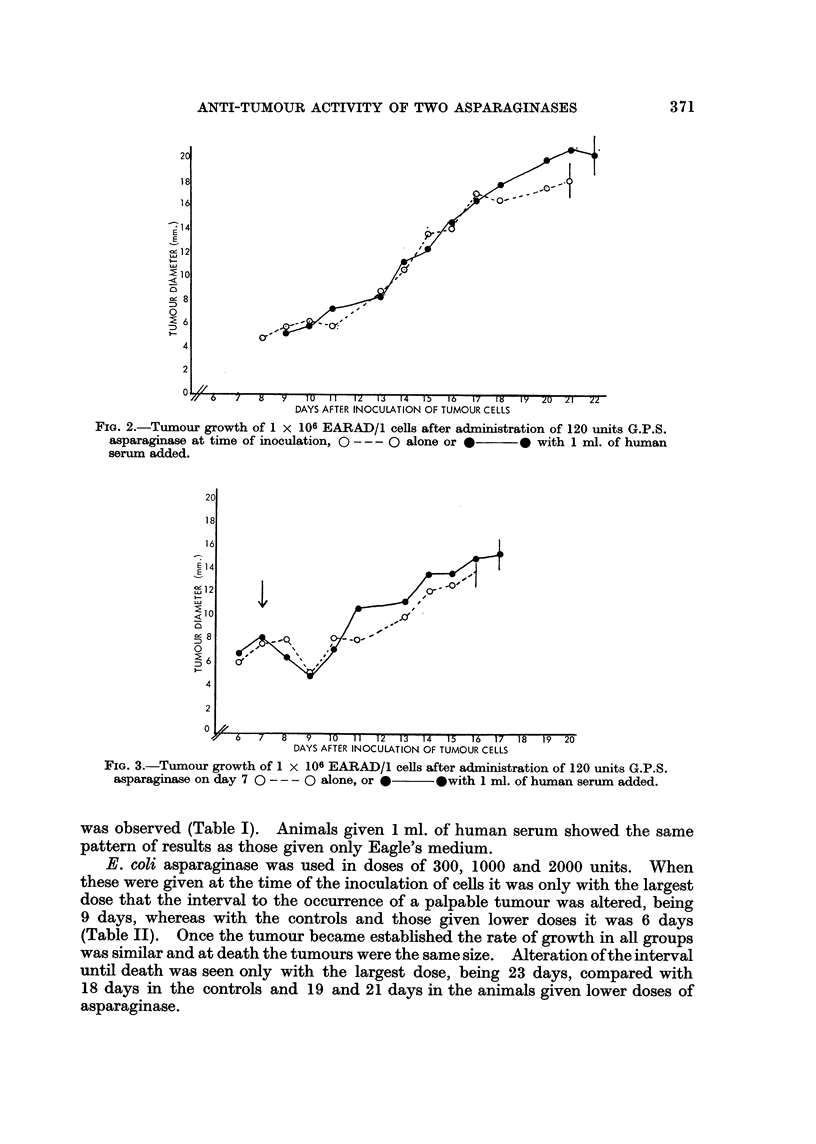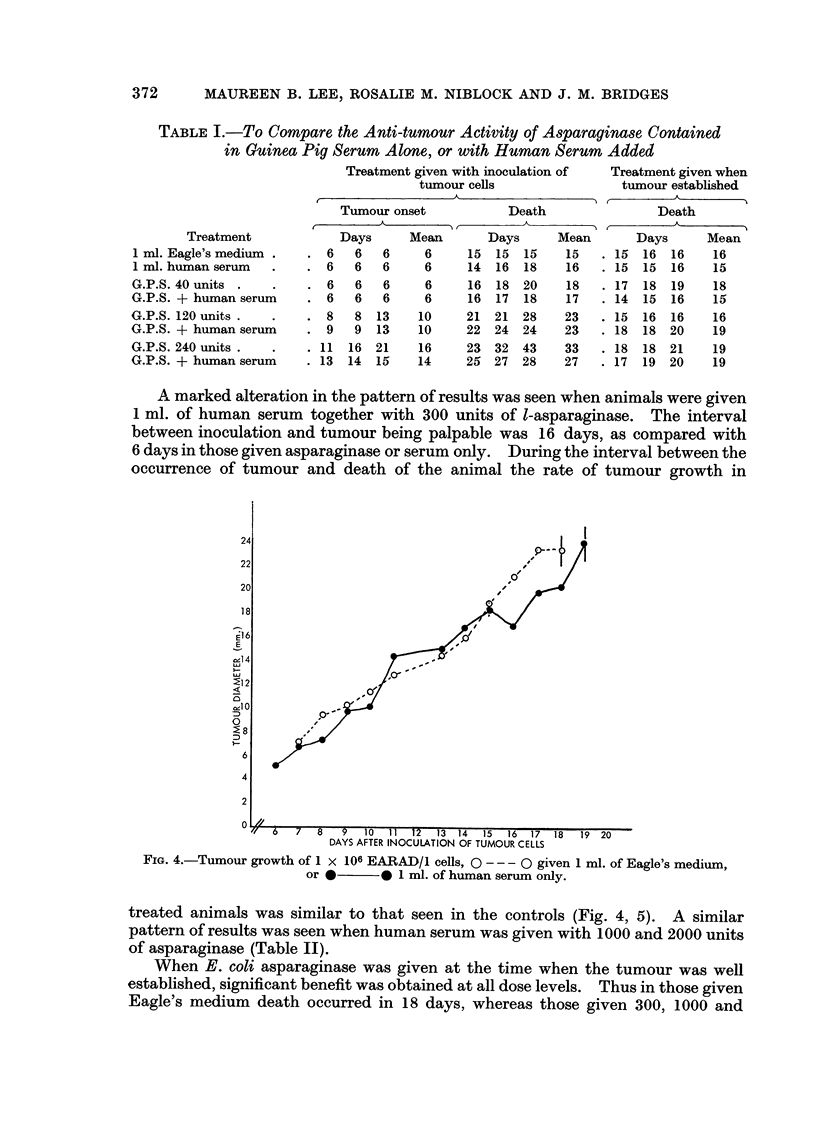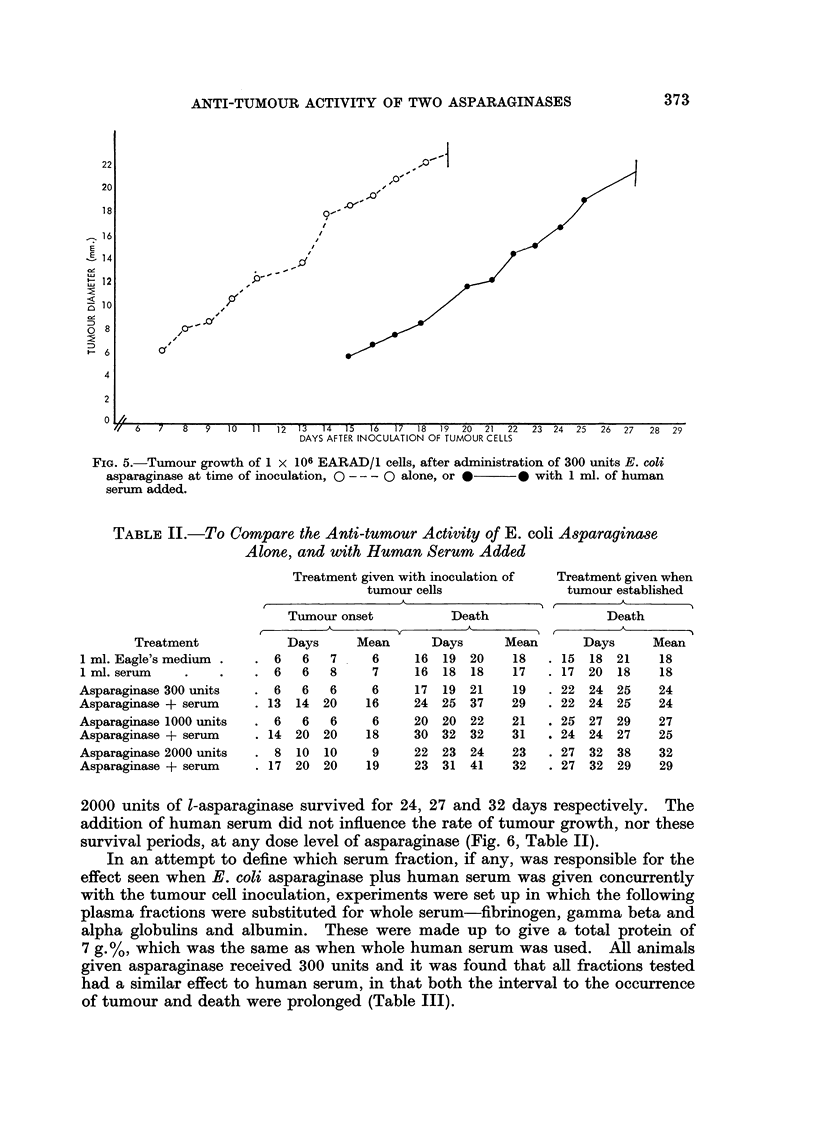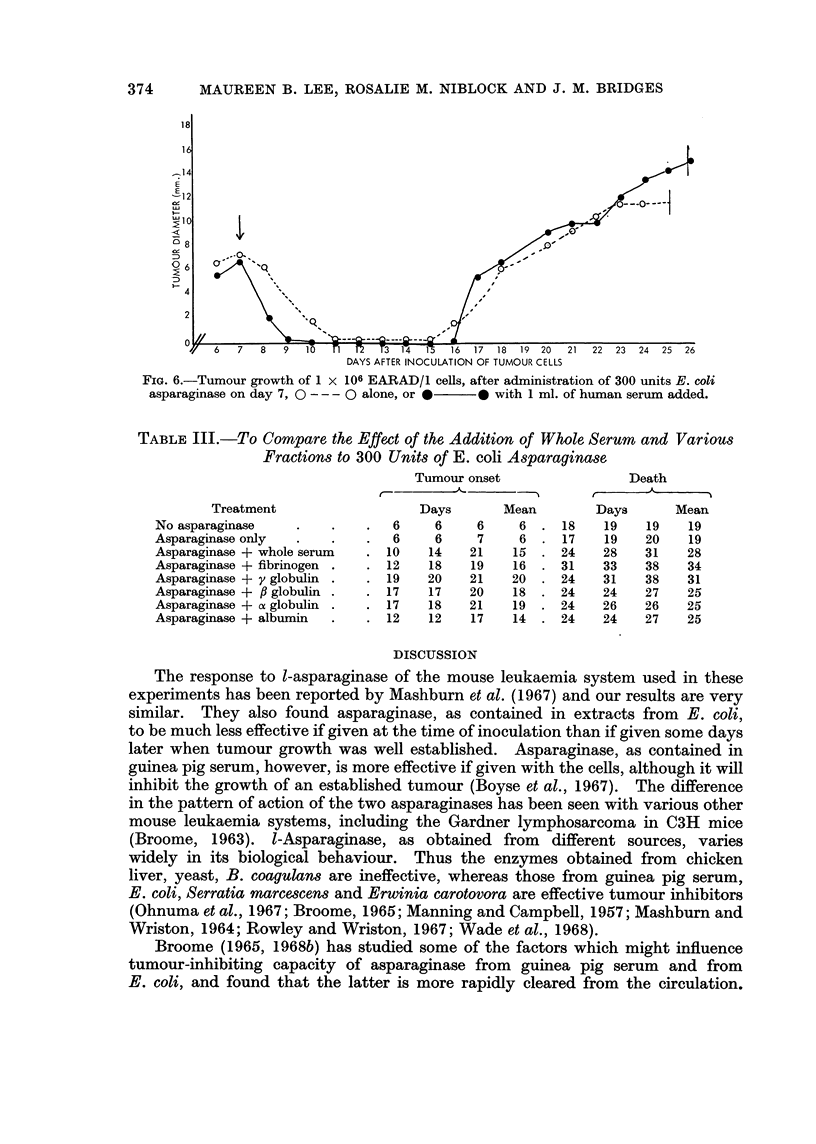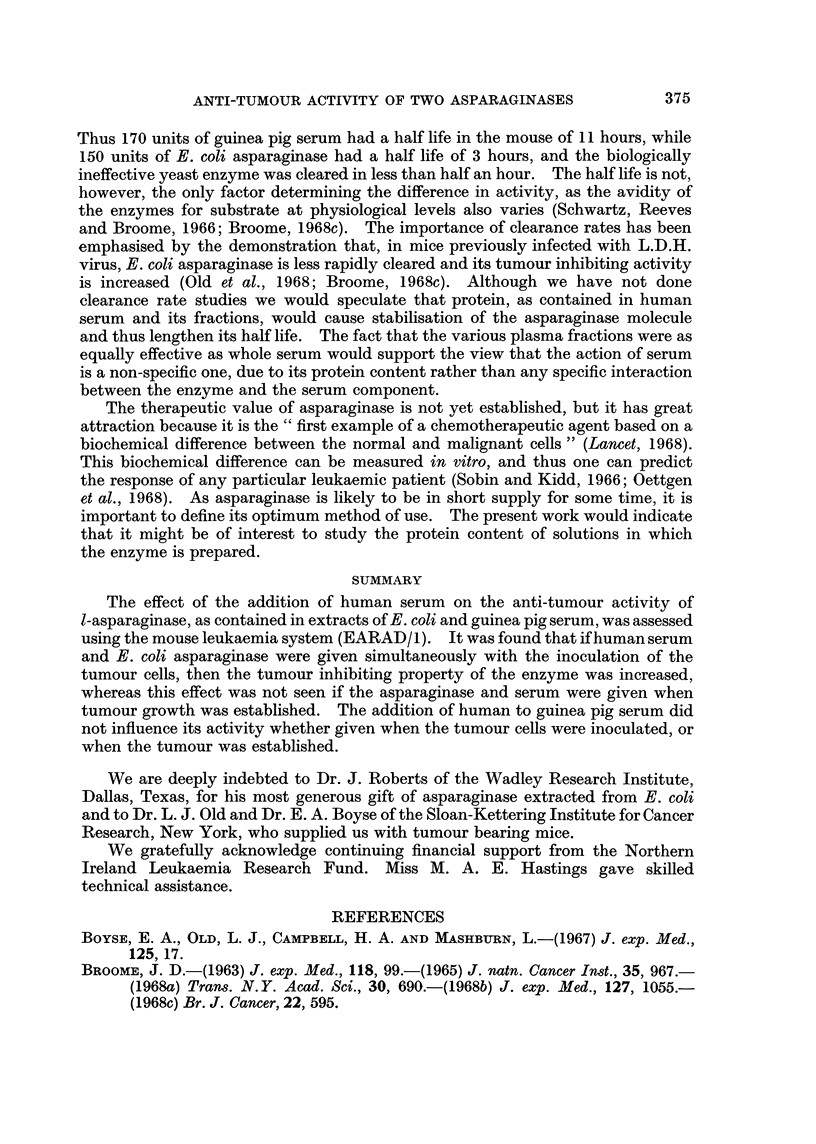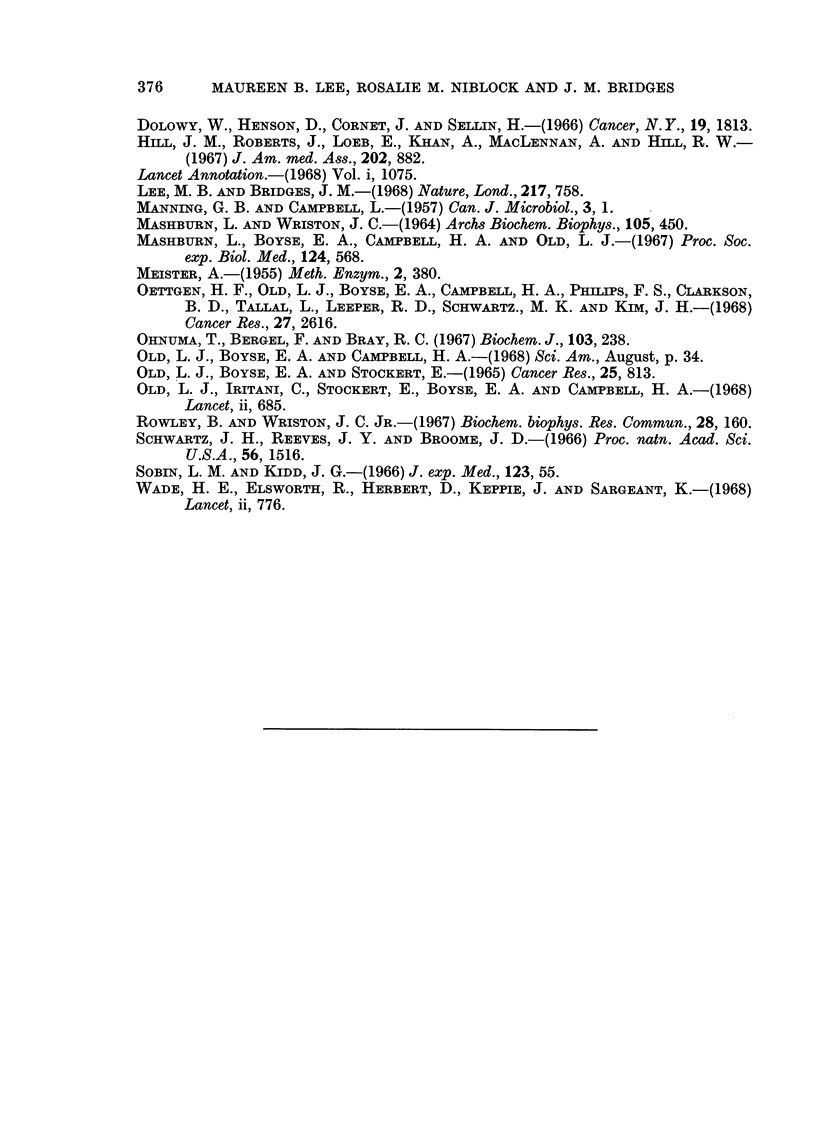# The influence of human serum on the anti-tumour activity of two L-asparaginases.

**DOI:** 10.1038/bjc.1969.48

**Published:** 1969-06

**Authors:** M. B. Lee, R. M. Niblock, J. M. Bridges


### THE INFLUENCE OF HUMAN SERUM ON THE ANTI-TUMOUR ACTIVITY OF TWO *L*-ASPARAGINASES

MAUREEN B. LEE, ROSALIE M. NIBLOCK AND J. M. BRIDGES

*From the Department of Clinical Pathology, Royal Victoria Hospital, Grosvenor Road, Belfast*

Received for publication January 24, 1969

THE ability of the enzyme *l*-asparaginase to inhibit the growth of certain rodent tumours has been extensively studied and the subject has been recently reviewed (Old, Boyse and Campbell, 1968; Broome, 1968*a*). Preliminary clinical trials (Dolowy *et al.*, 1967; Hill *et al.*, 1968; Oettgen *et al.*, 1968) indicate that it may be of value in some cases of human leukaemia, but its further evaluation is limited by the difficulty of extraction and preparation in a form suitable for therapeutic use.

The activity of *l*-asparaginase derived from *E. coli* has been shown to be enhanced *in vitro* by the addition of human and animal sera (Lee and Bridges, 1968). In this paper we report on the effect of the addition of human serum and its fractions on the tumour inhibiting property of *l*-asparaginase *in vivo.*

### MATERIALS AND METHODS

The assay of asparaginase was as previously described (Lee and Bridges, 1968) being based on that of Meister (1955). One unit of activity is that quantity of enzyme which releases 1 $\mu$mole of ammonia from *l*-asparagine per hour at the maximum rate.

The leukaemia used in this study, EARAD1 (Old, Boyse and Stockert, 1965), was induced by X-radiation in $(C57BL/6XA)F_1$ female mice and carried by us during the time of these experiments in transplant generations No. 137–156. Parent mice and hybrids were obtained from Jackson Laboratories. Spinner Modified Eagle's medium (Flow Laboratories) made 10% with horse serum was used for the suspension of cells during transplantation.

Pooled guinea pig serum from Dunkin Hartley guinea pigs was obtained by cardiac puncture and stored, without preservative, at $-20^\circ$ C. (Stayne Laboratories). The asparaginase activity was in the range 106–157 units per ml. The *E. coli* asparaginase used throughout the experiments was from a single batch of specific activity, 100 i.u./mg. of protein, obtained as a gift from the Wadley Research Institute, Dallas.

Human serum from normal subjects, Group $O^+$, was pooled and stored at $-20^\circ$ C. for up to six weeks without preservative. Freeze dried human plasma fractions (Baxter Hyland Laboratories) were reconstituted with normal saline.

### EXPERIMENTAL METHOD

Tumour cells from mice inoculated intraperitoneally some 10 days previously were aseptically harvested in Eagle's medium. Mice, in groups of 3, were inoculated subcutaneously with $1 \times 10^6$ tumour cells under an area of shaved skin. They received graded doses of guinea pig serum, equivalent to 40, 120 and 240 units, or *E. coli* asparaginase (300, 1000 and 2000 units), either alone or with 1 ml. of human serum added. Control animals received 1 ml. of Eagle's medium or 1 ml. of human serum only. These regimes were given either at the time of implantation of cells, or 7 days later when the tumour was well established, at which time the mean diameter was 7–8 mm. In experiments involving plasma fractions 1 dose of *E. coli* asparaginase (300 units) was used and the regime given only at the same time as the inoculation of the tumour cells.

To assess tumour growth, tumour diameter was measured in 3 directions and the mean taken. This observation was made daily following the occurrence of a palpable tumour. The intervals in days between inoculation of cells, occurrence of tumour and death were noted.

### RESULTS

In those mice given 1 ml. of Eagle's medium, tumours were palpable after 6 days and death occurred after 15 days, at which time the mean diameter of the tumour was 22 mm. (Fig. 1). If 40, 120 or 240 units of guinea pig serum asparaginase

FIG. 1.—Tumour growth of 1 × $10^6$ EARAD/1 cells ○ – – – ○ given 1 ml. of Eagle's medium or ●——● 1 ml. of human serum only.

were given, the intervals before the occurrence of the tumour were 6, 10 and 16 days respectively and death occurred at 18, 23 and 33 days; the rate of tumour growth and the ultimate size were not altered. This is shown for one animal given 120 units in Fig. 2. Identical doses of guinea pig serum asparaginase, given when the tumour was well established, caused temporary slight regression but the interval to death was not altered, being 18, 16 and 19 days respectively, as compared with those animals given only Eagle's medium, which died at 15 days (Fig. 3). If 1 ml. of human serum was added to the various doses of guinea pig serum, given either with the tumour cells or later, no difference in tumour inhibition

FIG. 2.—Tumour growth of 1 × $10^6$ EARAD/1 cells after administration of 120 units G.P.S. asparaginase at time of inoculation, ○ – – – ○ alone or ●———● with 1 ml. of human serum added.

FIG. 3.—Tumour growth of 1 × $10^6$ EARAD/1 cells after administration of 120 units G.P.S. asparaginase on day 7 ○ – – – ○ alone, or ●———●with 1 ml. of human serum added.

was observed (Table I). Animals given 1 ml. of human serum showed the same pattern of results as those given only Eagle's medium.

*E. coli* asparaginase was used in doses of 300, 1000 and 2000 units. When these were given at the time of the inoculation of cells it was only with the largest dose that the interval to the occurrence of a palpable tumour was altered, being 9 days, whereas with the controls and those given lower doses it was 6 days (Table II). Once the tumour became established the rate of growth in all groups was similar and at death the tumours were the same size. Alteration of the interval until death was seen only with the largest dose, being 23 days, compared with 18 days in the controls and 19 and 21 days in the animals given lower doses of asparaginase.

TABLE I.—*To Compare the Anti-tumour Activity of Asparaginase Contained in Guinea Pig Serum Alone, or with Human Serum Added*

| | Treatment given with inoculation of tumour cells | | | | | | | | Treatment given when tumour established | | | |
|---|---|---|---|---|---|---|---|---|---|---|---|---|
| | Tumour onset | | | | Death | | | | Death | | | |
| Treatment | Days | | | Mean | Days | | | Mean | Days | | | Mean |
| 1 ml. Eagle's medium | 6 | 6 | 6 | 6 | 15 | 15 | 15 | 15 | 15 | 16 | 16 | 16 |
| 1 ml. human serum | 6 | 6 | 6 | 6 | 14 | 16 | 18 | 16 | 15 | 15 | 16 | 15 |
| G.P.S. 40 units | 6 | 6 | 6 | 6 | 16 | 18 | 20 | 18 | 17 | 18 | 19 | 18 |
| G.P.S. + human serum | 6 | 6 | 6 | 6 | 16 | 17 | 18 | 17 | 14 | 15 | 16 | 15 |
| G.P.S. 120 units | 8 | 8 | 13 | 10 | 21 | 21 | 28 | 23 | 15 | 16 | 16 | 16 |
| G.P.S. + human serum | 9 | 9 | 13 | 10 | 22 | 24 | 24 | 23 | 18 | 18 | 20 | 19 |
| G.P.S. 240 units | 11 | 16 | 21 | 16 | 23 | 32 | 43 | 33 | 18 | 18 | 21 | 19 |
| G.P.S. + human serum | 13 | 14 | 15 | 14 | 25 | 27 | 28 | 27 | 17 | 19 | 20 | 19 |

A marked alteration in the pattern of results was seen when animals were given 1 ml. of human serum together with 300 units of *l*-asparaginase. The interval between inoculation and tumour being palpable was 16 days, as compared with 6 days in those given asparaginase or serum only. During the interval between the occurrence of tumour and death of the animal the rate of tumour growth in

FIG. 4.—Tumour growth of 1 × $10^6$ EARAD/1 cells, ○ - - - ○ given 1 ml. of Eagle's medium, or ●——● 1 ml. of human serum only.

treated animals was similar to that seen in the controls (Fig. 4, 5). A similar pattern of results was seen when human serum was given with 1000 and 2000 units of asparaginase (Table II).

When *E. coli* asparaginase was given at the time when the tumour was well established, significant benefit was obtained at all dose levels. Thus in those given Eagle's medium death occurred in 18 days, whereas those given 300, 1000 and

FIG. 5.—Tumour growth of 1 × $10^6$ EARAD/1 cells, after administration of 300 units *E. coli* asparaginase at time of inoculation, ○ – – – ○ alone, or ●———● with 1 ml. of human serum added.

TABLE II.—*To Compare the Anti-tumour Activity of* E. coli *Asparaginase Alone, and with Human Serum Added*

| | Treatment given with inoculation of tumour cells | | | | | | | | Treatment given when tumour established | | | |
|---|---|---|---|---|---|---|---|---|---|---|---|---|
| | Tumour onset | | | | Death | | | | Death | | | |
| Treatment | Days | | | Mean | Days | | | Mean | Days | | | Mean |
| 1 ml. Eagle's medium | 6 | 6 | 7 | 6 | 16 | 19 | 20 | 18 | 15 | 18 | 21 | 18 |
| 1 ml. serum | 6 | 6 | 8 | 7 | 16 | 18 | 18 | 17 | 17 | 20 | 18 | 18 |
| Asparaginase 300 units | 6 | 6 | 6 | 6 | 17 | 19 | 21 | 19 | 22 | 24 | 25 | 24 |
| Asparaginase + serum | 13 | 14 | 20 | 16 | 24 | 25 | 37 | 29 | 22 | 24 | 25 | 24 |
| Asparaginase 1000 units | 6 | 6 | 6 | 6 | 20 | 20 | 22 | 21 | 25 | 27 | 29 | 27 |
| Asparaginase + serum | 14 | 20 | 20 | 18 | 30 | 32 | 32 | 31 | 24 | 24 | 27 | 25 |
| Asparaginase 2000 units | 8 | 10 | 10 | 9 | 22 | 23 | 24 | 23 | 27 | 32 | 38 | 32 |
| Asparaginase + serum | 17 | 20 | 20 | 19 | 23 | 31 | 41 | 32 | 27 | 32 | 29 | 29 |

2000 units of *l*-asparaginase survived for 24, 27 and 32 days respectively. The addition of human serum did not influence the rate of tumour growth, nor these survival periods, at any dose level of asparaginase (Fig. 6, Table II).

In an attempt to define which serum fraction, if any, was responsible for the effect seen when *E. coli* asparaginase plus human serum was given concurrently with the tumour cell inoculation, experiments were set up in which the following plasma fractions were substituted for whole serum—fibrinogen, gamma beta and alpha globulins and albumin. These were made up to give a total protein of 7 g.%, which was the same as when whole human serum was used. All animals given asparaginase received 300 units and it was found that all fractions tested had a similar effect to human serum, in that both the interval to the occurrence of tumour and death were prolonged (Table III).

FIG. 6.—Tumour growth of 1 × $10^6$ EARAD/1 cells, after administration of 300 units *E. coli* asparaginase on day 7, ○ – – – ○ alone, or ●———● with 1 ml. of human serum added.

TABLE III.—*To Compare the Effect of the Addition of Whole Serum and Various Fractions to* 300 *Units of* E. coli *Asparaginase*

| Treatment | Tumour onset Days | | | Tumour onset Mean | Death Days | | | Death Mean |
|---|---|---|---|---|---|---|---|---|
| No asparaginase | 6 | 6 | 6 | 6 | 18 | 19 | 19 | 19 |
| Asparaginase only | 6 | 6 | 7 | 6 | 17 | 19 | 20 | 19 |
| Asparaginase + whole serum | 10 | 14 | 21 | 15 | 24 | 28 | 31 | 28 |
| Asparaginase + fibrinogen | 12 | 18 | 19 | 16 | 31 | 33 | 38 | 34 |
| Asparaginase + γ globulin | 19 | 20 | 21 | 20 | 24 | 31 | 38 | 31 |
| Asparaginase + β globulin | 17 | 17 | 20 | 18 | 24 | 24 | 27 | 25 |
| Asparaginase + α globulin | 17 | 18 | 21 | 19 | 24 | 26 | 26 | 25 |
| Asparaginase + albumin | 12 | 12 | 17 | 14 | 24 | 24 | 27 | 25 |

### DISCUSSION

The response to *l*-asparaginase of the mouse leukaemia system used in these experiments has been reported by Mashburn *et al.* (1967) and our results are very similar. They also found asparaginase, as contained in extracts from *E. coli*, to be much less effective if given at the time of inoculation than if given some days later when tumour growth was well established. Asparaginase, as contained in guinea pig serum, however, is more effective if given with the cells, although it will inhibit the growth of an established tumour (Boyse *et al.*, 1967). The difference in the pattern of action of the two asparaginases has been seen with various other mouse leukaemia systems, including the Gardner lymphosarcoma in C3H mice (Broome, 1963). *l*-Asparaginase, as obtained from different sources, varies widely in its biological behaviour. Thus the enzymes obtained from chicken liver, yeast, *B. coagulans* are ineffective, whereas those from guinea pig serum, *E. coli*, *Serratia marcescens* and *Erwinia carotovora* are effective tumour inhibitors (Ohnuma *et al.*, 1967; Broome, 1965; Manning and Campbell, 1957; Mashburn and Wriston, 1964; Rowley and Wriston, 1967; Wade *et al.*, 1968).

Broome (1965, 1968*b*) has studied some of the factors which might influence tumour-inhibiting capacity of asparaginase from guinea pig serum and from *E. coli*, and found that the latter is more rapidly cleared from the circulation.

Thus 170 units of guinea pig serum had a half life in the mouse of 11 hours, while 150 units of *E. coli* asparaginase had a half life of 3 hours, and the biologically ineffective yeast enzyme was cleared in less than half an hour. The half life is not, however, the only factor determining the difference in activity, as the avidity of the enzymes for substrate at physiological levels also varies (Schwartz, Reeves and Broome, 1966; Broome, 1968*c*). The importance of clearance rates has been emphasised by the demonstration that, in mice previously infected with L.D.H. virus, *E. coli* asparaginase is less rapidly cleared and its tumour inhibiting activity is increased (Old *et al.*, 1968; Broome, 1968*c*). Although we have not done clearance rate studies we would speculate that protein, as contained in human serum and its fractions, would cause stabilisation of the asparaginase molecule and thus lengthen its half life. The fact that the various plasma fractions were as equally effective as whole serum would support the view that the action of serum is a non-specific one, due to its protein content rather than any specific interaction between the enzyme and the serum component.

The therapeutic value of asparaginase is not yet established, but it has great attraction because it is the " first example of a chemotherapeutic agent based on a biochemical difference between the normal and malignant cells " (*Lancet*, 1968). This biochemical difference can be measured *in vitro*, and thus one can predict the response of any particular leukaemic patient (Sobin and Kidd, 1966; Oettgen *et al.*, 1968). As asparaginase is likely to be in short supply for some time, it is important to define its optimum method of use. The present work would indicate that it might be of interest to study the protein content of solutions in which the enzyme is prepared.

SUMMARY

The effect of the addition of human serum on the anti-tumour activity of *l*-asparaginase, as contained in extracts of *E. coli* and guinea pig serum, was assessed using the mouse leukaemia system (EARAD/1). It was found that if human serum and *E. coli* asparaginase were given simultaneously with the inoculation of the tumour cells, then the tumour inhibiting property of the enzyme was increased, whereas this effect was not seen if the asparaginase and serum were given when tumour growth was established. The addition of human to guinea pig serum did not influence its activity whether given when the tumour cells were inoculated, or when the tumour was established.

We are deeply indebted to Dr. J. Roberts of the Wadley Research Institute, Dallas, Texas, for his most generous gift of asparaginase extracted from *E. coli* and to Dr. L. J. Old and Dr. E. A. Boyse of the Sloan-Kettering Institute for Cancer Research, New York, who supplied us with tumour bearing mice.

We gratefully acknowledge continuing financial support from the Northern Ireland Leukaemia Research Fund. Miss M. A. E. Hastings gave skilled technical assistance.

### REFERENCES

Boyse, E. A., Old, L. J., Campbell, H. A. and Mashburn, L.—(1967) *J. exp. Med.*, **125**, 17.

Broome, J. D.—(1963) *J. exp. Med.*, **118**, 99.—(1965) *J. natn. Cancer Inst.*, **35**, 967.—(1968*a*) *Trans. N.Y. Acad. Sci.*, **30**, 690.—(1968*b*) *J. exp. Med.*, **127**, 1055.—(1968*c*) *Br. J. Cancer*, **22**, 595.

Dolowy, W., Henson, D., Cornet, J. and Sellin, H.—(1966) *Cancer, N.Y.*, **19**, 1813.
Hill, J. M., Roberts, J., Loeb, E., Khan, A., MacLennan, A. and Hill, R. W.—(1967) *J. Am. med. Ass.*, **202**, 882.
*Lancet Annotation.*—(1968) Vol. i, 1075.
Lee, M. B. and Bridges, J. M.—(1968) *Nature, Lond.*, **217**, 758.
Manning, G. B. and Campbell, L.—(1957) *Can. J. Microbiol.*, **3**, 1.
Mashburn, L. and Wriston, J. C.—(1964) *Archs Biochem. Biophys.*, **105**, 450.
Mashburn, L., Boyse, E. A., Campbell, H. A. and Old, L. J.—(1967) *Proc. Soc. exp. Biol. Med.*, **124**, 568.
Meister, A.—(1955) *Meth. Enzym.*, **2**, 380.
Oettgen, H. F., Old, L. J., Boyse, E. A., Campbell, H. A., Philips, F. S., Clarkson, B. D., Tallal, L., Leeper, R. D., Schwartz., M. K. and Kim, J. H.—(1968) *Cancer Res.*, **27**, 2616.
Ohnuma, T., Bergel, F. and Bray, R. C. (1967) *Biochem. J.*, **103**, 238.
Old, L. J., Boyse, E. A. and Campbell, H. A.—(1968) *Sci. Am.*, August, p. 34.
Old, L. J., Boyse, E. A. and Stockert, E.—(1965) *Cancer Res.*, **25**, 813.
Old, L. J., Iritani, C., Stockert, E., Boyse, E. A. and Campbell, H. A.—(1968) *Lancet*, ii, 685.
Rowley, B. and Wriston, J. C. Jr.—(1967) *Biochem. biophys. Res. Commun.*, **28**, 160.
Schwartz, J. H., Reeves, J. Y. and Broome, J. D.—(1966) *Proc. natn. Acad. Sci. U.S.A.*, **56**, 1516.
Sobin, L. M. and Kidd, J. G.—(1966) *J. exp. Med.*, **123**, 55.
Wade, H. E., Elsworth, R., Herbert, D., Keppie, J. and Sargeant, K.—(1968) *Lancet*, ii, 776.